# An overview of video recommender systems: state-of-the-art and research issues

**DOI:** 10.3389/fdata.2023.1281614

**Published:** 2023-10-30

**Authors:** Sebastian Lubos, Alexander Felfernig, Markus Tautschnig

**Affiliations:** ^1^Applied Software Engineering & AI Research Group, Institute of Software Technology, Graz University of Technology, Graz, Austria; ^2^Streamdiver GmbH, Klagenfurt am Wörthersee, Austria

**Keywords:** video recommender systems, collaborative filtering, content-based recommendation, hybrid recommenders, group recommenders, decision-making, overview, research challenges

## Abstract

Video platforms have become indispensable components within a diverse range of applications, serving various purposes in entertainment, e-learning, corporate training, online documentation, and news provision. As the volume and complexity of video content continue to grow, the need for personalized access features becomes an inevitable requirement to ensure efficient content consumption. To address this need, recommender systems have emerged as helpful tools providing personalized video access. By leveraging past user-specific video consumption data and the preferences of similar users, these systems excel in recommending videos that are highly relevant to individual users. This article presents a comprehensive overview of the current state of *video recommender systems (VRS)*, exploring the algorithms used, their applications, and related aspects. In addition to an in-depth analysis of existing approaches, this review also addresses unresolved research challenges within this domain. These unexplored areas offer exciting opportunities for advancements and innovations, aiming to enhance the accuracy and effectiveness of personalized video recommendations. Overall, this article serves as a valuable resource for researchers, practitioners, and stakeholders in the video domain. It offers insights into cutting-edge algorithms, successful applications, and areas that merit further exploration to advance the field of video recommendation.

## 1. Introduction

*Recommender systems (RS)* support various decision-making scenarios ranging from the recommendation of simple items, such as books or movies, to more complex ones, like financial services and digital equipment (Ricci et al., 2011). Among these applications, *movie recommender systems* stand out as a pioneering example, suggesting movies that users may find interesting to watch (Harper and Konstan, 2015). These movie recommenders are a specific category within *video recommender systems (VRS)*, which are gaining significant attention in entertainment, as well as industrial contexts, due to the rapidly increasing number of available video items.

Popular video platforms, for example, YOUTUBE^1^ and NETFLIX,^2^ integrate recommendation technologies to enhance user experience by suggesting videos from their huge catalogs that are likely to align with users' personal interests and preferences (Davidson et al., 2010; Gomez-Uribe and Hunt, 2016). From an economic perspective, these platforms aim to attract and retain customers, increasing the retention rate through effective content recommendations (Gomez-Uribe and Hunt, 2016). For instance, around two-thirds of the content streamed on NETFLIX originates from recommendations featured on the entry page (Gomez-Uribe and Hunt, 2016). Moreover, empirical studies have demonstrated that video recommendations can capture a user's attention toward specific topics and consequently increase the popularity of particular videos (Wu et al., 2019), emphasizing the power of this technology.

Several reviews related to video recommendations have been published in the past years. In Véras et al. (2015) recommender systems in the television domain are covered, including content related to TV shows. In Wang and Zhao (2022), an in-depth analysis of affective video recommender systems, i.e., systems that integrate human-like capabilities of observation, interpretation, and generation of affect features, like, emotions and mood, is provided. A broader overview of multimedia item recommenders, encompassing audio, images, and videos, is presented in Deldjoo et al. (2022), focusing on methods for feature extraction and integration of multimedia data as side information in recommenders. In Jayalakshmi et al. (2022), a literature review on movie recommender systems is provided, discussing algorithmic commonalities and recent publications in this domain.

While those related reviews specialize in specific video-related recommender aspects, our overview provides a concise summary of video item recommendations, serving as a comprehensible summary of the state-of-the-art for practitioners and researchers in this area. This overview should enhance understanding of the various technical approaches within this field and their applications. Additionally, it identifies open issues that should be addressed in future research to further develop the field.

The article is structured as follows: In Section 2, we outline the analysis method employed in our literature review. In Section 3, we conduct an in-depth analysis of the existing literature on VRS, categorizing it based on different fundamental approaches of recommender systems and the technologies utilized. Following that, in Section 4, we discuss the findings and offer insights to comprehend which approaches excel in various recommendation scenarios. Additionally, we address future research considerations and discuss unresolved issues. Finally, the article concludes in Section 5.

The major contributions of this article can be summarized as follows: *Firstly*, we present an extensive overview of the current state-of-the-art in VRS, covering research developments from the past decades. *Secondly*, we provide valuable guidance for selecting suitable recommendation approaches based on individual scenarios. *Thirdly*, we engage in a comprehensive discussion of open research issues, highlighting the potential for future work in this evolving field of research.

## 2. Methods

The main objective of this article is to provide an overview of state-of-the-art video recommender systems to increase understanding of this topic, derive guidance for choosing appropriate approaches, and identify issues for future research. In this context, we include recommender systems where the recommended items are *videos*, independent of the domain. This includes entertainment, e.g., movies or videos on social networks, as well as video advertisements, learning videos, news videos, and others.

Our analysis of related work is based on a bibliographic review method. As an initial step, we collected and reviewed existing publications on VRS over the last 20 years. The search for related papers was performed on the basis of different keywords, including, “*video recommender systems”*, “*video recommender”*, “*video recommendation”*, “*movie recommender systems”*, “*movie recommender”*, and “*movie recommendation”*. With these, queries were triggered in the digital libraries of ACM,^3^ GOOGLE SCHOLAR,^4^ RESEARCHGATE,^5^ SCIENCE DIRECT,^6^ and SPRINGER LINK.^7^

Following the review, publications were categorized by their recommendation approach (content-based, collaborative, hybrid, and group recommendation), and further divided into subcategories of different applied algorithms. The results are outlined below. The topic of video content representation, which is relevant for content-based and many hybrid recommender approaches, is summarized in a separate section. From these findings, guidance in selecting appropriate technologies is derived and open topics for future research are identified.

## 3. Video recommender systems

**Figure 1:**
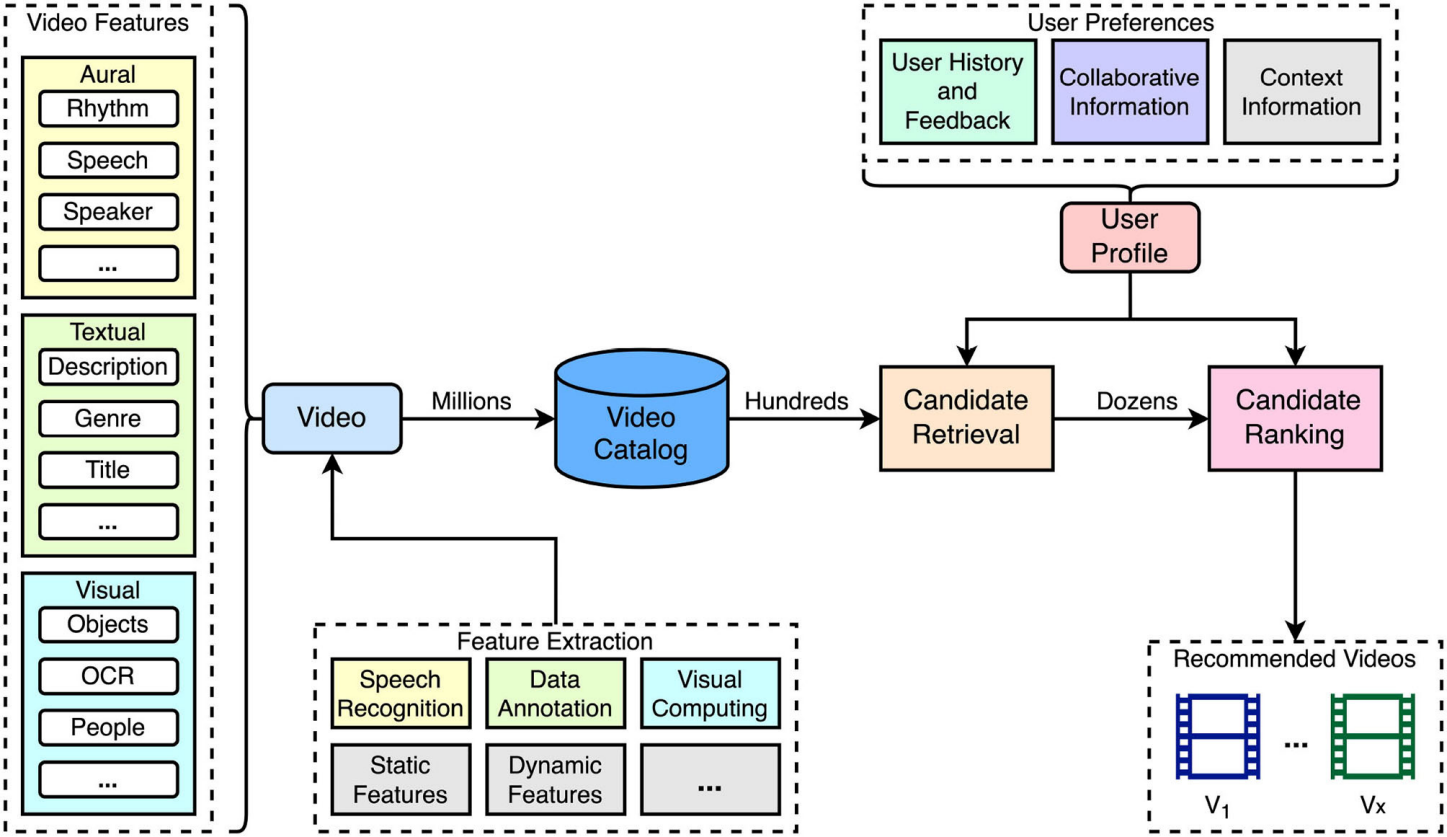
Overview of the pipeline used in video recommender systems. Typically, videos are indexed in the catalog using feature descriptions that are either automatically extracted or added manually (see Section 3.1). Using the videos in the catalog, personalized recommendations are retrieved in a two-step phase by identifying candidates and ranking them based on the generated user profile describing their preferences.

Video recommender systems suggest videos to users based on their individual preferences. An overview of a typical pipeline used for video recommendation is illustrated in Figure 1. A specialty for recommendations in the video domain is the representation of content in terms of features that are automatically extracted or manually added. Videos offer a rich variety of different features that can be used to describe their content. Details on content representation are discussed in Section 3.1.

Similar to recommendations in other item domains, dealing with a large catalog of videos can lead to performance issues. To address this, a common approach is to split the computation in a *retrieval* and *ranking* phase (Davidson et al., 2010; Covington et al., 2016; Gomez-Uribe and Hunt, 2016). The retrieval phase reduces the number of candidates to a reasonable number using a relatively fast analysis. In the ranking phase, the remaining candidates are ordered by relevance using more precise but often slower algorithms. This two-step strategy enables efficient video recommendations from extensive catalogs within an acceptable time. Both steps consider a user profile generated from information, such as the user history of consumed videos, provided feedback, information of similar users, and the current user context.

The variability of VRS applications can be illustrated by taking NETFLIX as an example (Gomez-Uribe and Hunt, 2016). The platform uses a *personalized video ranker (PVR)* algorithm to order its video catalog based on user profiles, video popularity, and temporal viewing trends. Different algorithms are applied on top for various purposes: (1) Identifying the most relevant items from the catalog for each user. (2) Ordering videos users have started watching. (3) Unpersonalized prediction of short-term temporal trends for events like Halloween or Christmas, or unplanned incidents, such as a hurricane or other natural catastrophes currently populated by the news. (4) Recommending videos with similar content. (5) Enhancing content presentation by selecting thumbnails and presented metadata. Furthermore, NETFLIX employs a *page generation* algorithm to define the selection and ordering of rows presented in the UI. It considers that one account is mostly used by multiple users, e.g., family members, aiming for a diverse content presentation that is relevant to each user in front of the screen.

**Figure 2:**
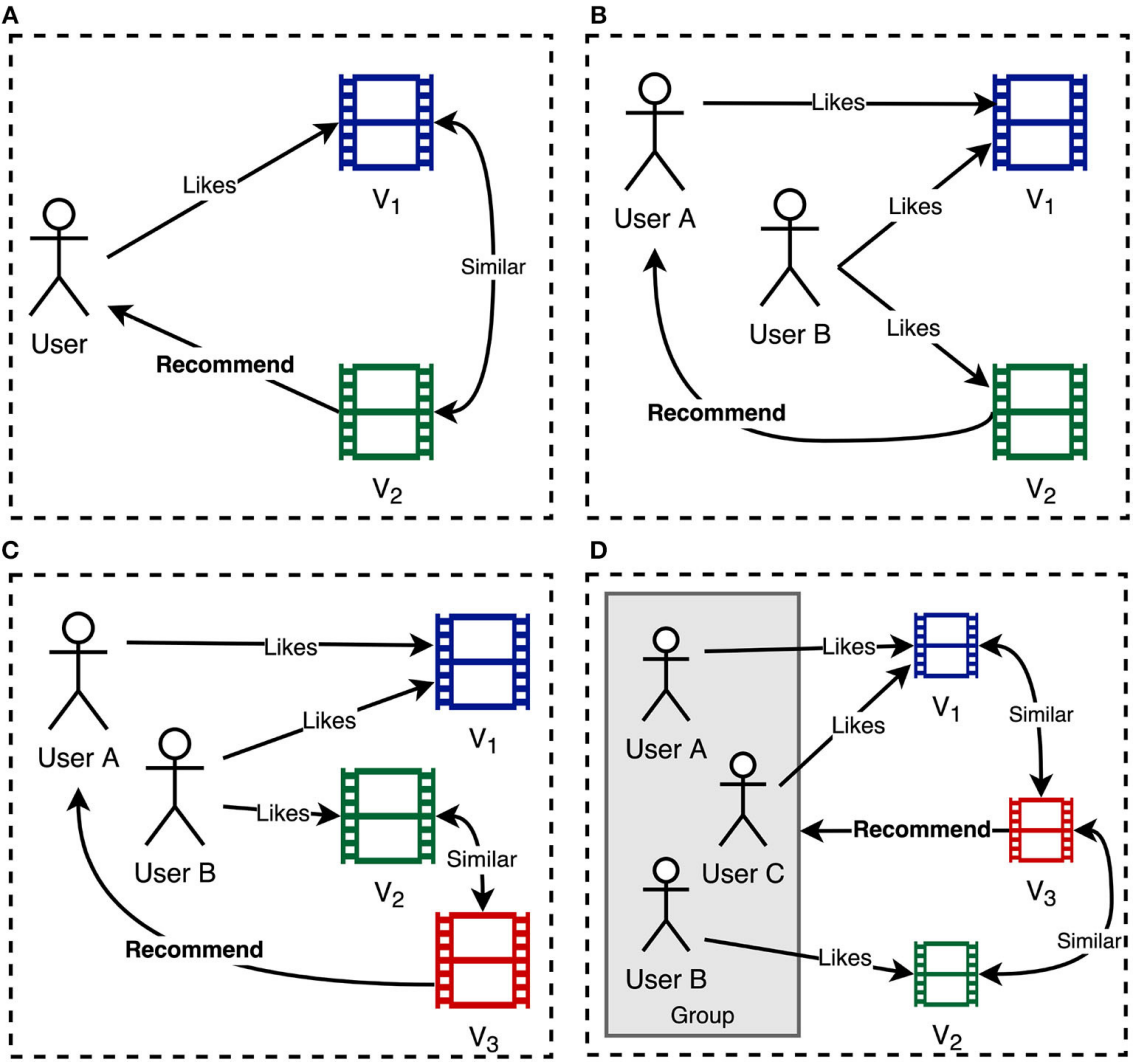
Simplified overview of different recommendation approaches. **(A)** Content-based recommendation. **(B)** Collaborative filtering. **(C)** Hybrid recommendation. **(D)** Group recommendation.

In the following, the literature on VRS is discussed. Foremost, the methods used to represent the content of videos are discussed. Subsequently, publications are categorized by the applied recommendation approach, including *content-based recommendation, collaborative filtering (CF), hybrid recommendation*, and *group recommendation*. In Figure 2, a simplified overview of the different approaches is shown. While content-based recommendation (see Figure 2A) recommends videos to a user based on their similarity, collaborative filtering (see Figure 2B) exploits the knowledge of users with similar interests. Hybrid recommenders (see Figure 2C) combine different approaches to generate recommendations. While the aforementioned approaches focus on recommending items to individual users, group recommenders (see Figure 2D) try to suggest videos that are in line with the preferences of a group consisting of multiple persons.

### 3.1. Content representation

Video recommenders differ notably from those in many other domains, e.g., shopping, due to the nature of the items being recommended. Unlike structured features like color, brand, category, or price that describe shopping items, video content descriptions encompass more possibilities due to their *multimodality*. Videos consist of three modalities: (1) *Aural* (audio information), (2) *Visual* (visual frames), and (3) *Textual* (textual descriptions and metadata), which can be expressed in varying degrees of semantic detail. This characteristic makes videos *multi-modal*, as they include all three modalities, whereas a music piece without lyrics is *uni-modal*, as it only features aural elements (Deldjoo, 2020).

Based on the classification in Deldjoo (2020), video features can be categorized into groups based on their modality and semantic expressiveness: (1) *Low-level* features describe the raw signal of a video, representing its stylistic properties. (2) *Mid-level* features require interpretation knowledge and are derived from low-level features, representing syntactic features. (3) *High-level* features resemble human interpretation of the content, providing a semantic description. Table 1 presents an overview of these categorized features, enabling the classification of VRS based on the features they use for computing recommendations.

**Table 1:** Multimedia features categorized by their expressiveness and modality.

**Hierarchy**	**Modalities**		
	**Aural**	**Textual**	**Visual**
Low-level	Beat, frequency, loudness, intensity, pitch, timbre	n-grams, tokens	Colors, contours, edges, key points, keyframes, motions, shapes, textures
Mid-level	Note onsets, patterns, rhythm, tempo	Paragraphs, sentences, term-frequencies, transcript	Actions, interactions, objects, people, scenes, shots, scenes
High-level	Events, mood, speech, speaker, story	Comments, description, genre, events, keywords, key phrases, named entities, sentiment, story, tags, title, topic, writing style	Concept, emotion, message, language, speaker, structure

The table extends the one provided in Deldjoo (2020).

Content descriptions in the video domain can be manually created or automatically extracted. Manual features typically include a title, a short description, and tags. For movies, databases like *Internet Movie Database (IMDb)*^8^ and *Open Movie Database (OMDB)*^9^ provide structured metadata including actors, genres, plots, and more. Another option is the extension with *semantic web data*, illustrated in Hopfgartner and Jose (2010), which leverages LINKED OPEN DATA CLOUD^10^ for content description enrichment.

Automatically extracted features in the video domain offer diverse options in semantic expressiveness and modalities. A common technique is the conversion into *embeddings*, representing words as numerical vectors in a lower-dimensional space, preserving item feature information (Huang et al., 2019). This approach provides a compact representation and enables mathematical operations on the embeddings.

Videos share similarities in processing with other multimedia items like audio and images. Image processing methods can be applied to video frames for visual feature retrieval, while audio processing techniques analyze the audio track. Yet, videos offer additional temporal attributes, enabling action and motion recognition over time. For more details on fundamental extraction methods for multimedia items, refer to Deldjoo et al. (2022). In this overview, we focus on algorithmic approaches and applications of video recommenders, utilizing both manually created and automatically extracted features.

Table 2 summarizes the content modalities used in various video domains. The table shows that research on VRS with content representations predominantly focuses on *Movies and Series* and videos within *Social Networks*. Reasons might be the significant user base and availability of datasets in these domains (see Section 3.6.4). Initiatives like the *Netflix Prize* have contributed to this emphasis by providing real-life data to improve movie recommendation accuracy (Bennett and Lanning, 2007).

**Table 2:** Content representations in VRS classified by domain and used feature modalities.

**Domain**	**Features**	**References**
Advertisement	Textual	Kaklauskas et al., 2018; Kim et al., 2021
Education	Textual	Chantanurak et al., 2016; Kimoto et al., 2016; Tavakoli et al., 2020; Leite et al., 2022
Movies and Series	Aural	Deldjoo et al., 2018a; Rimaz et al., 2021; Chakder et al., 2022; Pingali et al., 2022; Mondal et al., 2023
	Textual	Öztürk and Kesim Cicekli, 2011; Zhu et al., 2013; Vizine Pereira and Hruschka, 2015; Wang et al., 2015, 2021; Gomez-Uribe and Hunt, 2016; Elahi et al., 2017; Lu et al., 2017; Wei et al., 2017; Liu et al., 2019b; Kvifte et al., 2021; Zhuo et al., 2021; Chakder et al., 2022; Pingali et al., 2022; Mondal et al., 2023
	Visual	Zhu et al., 2013; Deldjoo et al., 2016, 2018a,b; Elahi et al., 2017, 2020, 2021; Hazrati and Elahi, 2021; Kvifte et al., 2021; Wang et al., 2021; Chakder et al., 2022; Pingali et al., 2022; Mondal et al., 2023
News	Aural	Luo et al., 2008
	Textual	Luo et al., 2008; Hopfgartner and Jose, 2010
	Visual	Luo et al., 2008
Social Networks	Aural	Mei et al., 2007, 2011; Niu et al., 2013; Lee and Abu-El-Haija, 2017; Liu et al., 2019a; Du et al., 2022; Yi et al., 2022
	Textual	Mei et al., 2007, 2011; Wu et al., 2008; Davidson et al., 2010; Cui et al., 2014; Covington et al., 2016; Abbas et al., 2017; Gao et al., 2017; Chen et al., 2018, 2021; Li et al., 2019; Liu et al., 2019a; Jiang et al., 2020; Tang et al., 2020; Du et al., 2022; Gong et al., 2022; Yi et al., 2022; Song et al., 2023; Xiao et al., 2023
	Visual	Mei et al., 2007, 2011; Niu et al., 2013; Roy and Guntuku, 2016; Gao et al., 2017; Lee and Abu-El-Haija, 2017; Chen et al., 2018, 2021; Li et al., 2019; Liu et al., 2019a; Ma et al., 2019; Du et al., 2022; Yi et al., 2022
Sports	Textual	Sanchez et al., 2012
		Visual	Ramezani and Yaghmaee, 2016

Publications may appear multiple times if more than one feature modality is used.

Based on the summary, video recommender system research has employed diverse modalities to represent video content, revealing certain trends. Aural features were infrequently used, and when applied, were often combined with textual or visual features. This implies that sole reliance on aural features might lack accuracy. Visual features were prevalent, especially in entertainment domains, where visuals are significant. Textual features were widely adopted across domains, likely due to the ability to reuse technical approaches from other domains and the rich information they provide, particularly in educational videos where facts are more relevant than visual aspects.

Generating appropriate suitable video representations is crucial in video recommendation and has been extensively studied. The study in Elahi et al. (2017) focused on the *semantic gap*, which refers to the difference between various representations of the same item. The study evaluated various video representations and found that both low-level stylistic features (e.g., brightness and contrast) and high-level semantic concepts (e.g., genre and actors) contribute to accurate recommendations. Combining these features through a multi-modal approach showed potential for improving accuracy.

A related study found similar results with automatically extracted aural and visual features (Deldjoo et al., 2018a). Aural features included short audio segment characteristics (*Block-Level-Features*) and low-dimensional representations of acoustic signals (*I-Vector Features*). Visual features included *Aesthetic Visual Features (AVF)*, categorized by color, texture, and objects, as well as high-level features extracted with *Deep Neural Networks (DNN)*. Utilizing multi-modal representations with weighted aggregation again demonstrated the potential for improving accuracy.

The positive impact of multi-modal representations with automatically extracted aural and visual features was also observed in Lee and Abu-El-Haija (2017), where optimization options for embedding representations were explored. Increasing the output feature size of embeddings, utilizing deeper models, enhancing the capacity of the first hidden layer, and applying late fusion of aural and visual features led to more accurate recommendations. The representations were found to capture the semantic features of items, despite the features themselves not being inherently semantic. Moreover, the representations proved effective in accurately recommending videos on the same topic but in different languages. The possibility to predict descriptive tags for videos from low-level visual features was described (Elahi et al., 2020), confirming the possibility to generate features with semantic meaning from unsemantic data.

In Pingali et al. (2022), a multi-modal content representation approach for movies is proposed, which involves concatenating feature embeddings from aural and visual features, textual descriptions, and other metadata to create a vector representation of the video in a vector space. Those unsupervised methods for generating content representations help address the challenge of cold start, where limited or no initial information is available and reduce manual effort at the same time (Hazrati and Elahi, 2021).

The study in Deldjoo et al. (2016) highlights essential findings regarding video representation. Low-level visual features from movie trailers accurately capture the full movie's essence, enabling performance tuning with smaller samples. Automatic extraction of visual features addresses missing content descriptions for competitive accuracy in recommendations. However, combining various features might reduce accuracy due to a lack of correlation between them. Subsequent research in Deldjoo et al. (2018b) validates this, showing that maximizing pairwise correlation through feature fusion does not enhance accuracy, suggesting that stylistically similar movies might not share semantic commonalities.

Each visual feature has different capabilities to capture the video content appropriately and thus can contribute differently to the creation of recommendations (Hazrati and Elahi, 2021). Combining features can enhance recommendation accuracy if their information is not contradicting. The same is true for the aural features of videos (Rimaz et al., 2021).

High-level visual features such as faces, objects, and recognized celebrities were automatically extracted in Elahi et al. (2021), to create vector representations for videos using a combination of *term frequency-inverse document frequency (TF-IDF)* (Sammut and Webb, 2010) and *word2vec* (Mikolov et al., 2013). TF-IDF is a statistical measure that reflects the importance of terms within a document or catalog, while word2vec describes a DNN technique used in *Natural Language Processing (NLP)* to learn word relationships. This representation incorporating semantic features allows for human comprehension of recommendations and offers the potential to explain why a video is suggested.

*Restricted Boltzmann Machines (RBM)* are a type of *neural network (NN)* used in Hazrati and Elahi (2021) to learn the latent representation of videos in a feature space. Visual features are employed for model training, capturing complex connections in the input features. The model assigns different weights to individual input features, reflecting their representativeness of the video content.

An alternative approach for content representation is to classify videos by topic using extracted features as input. For instance, in Luo et al. (2008), multi-modal features are synchronized to learn topic representations for news videos, while Zhu et al. (2013) introduces a topic-modeling approach for movies.

A special task of VRS is the recommendation of *micro videos* (sometimes *short videos*), commonly found on social network platforms, like TIKTOK.^11^ These videos have a small duration (usually seconds to minutes) and limited textual descriptions, requiring systems to rely on automatically extracted features for their recommendations.

*Multi-Modal Graph Contrastive Learning (MMGCL)* is introduced in Yi et al. (2022) to learn multi-modal representations for micro videos. This self-supervised method employs augmentation techniques and negative sampling to achieve accurate representations, considering the correlation between different modalities. Similarly, in Du et al. (2022), the modality correlation is explored using a *Cross-modal Graph Neural Network* to encode and aggregate cross-model information, enabling the creation of modality-aware representations for users and micro videos. The self-supervised learning approach used is *Cross-modal Mutual Information Fusion*, which captures the correlation between video modalities.

The *VideoReach* system (Mei et al., 2007, 2011), addresses the integration of multi-modal features for video representation. It combines manually crafted and automatically extracted aural, textual, and visual features, mapping them to textual descriptions for compatibility with textual recommendation methods. The system assigns predetermined weights to feature types, focusing more on textual features due to their rich information content. These weights are individually adjusted based on user feedback, measured through the *Click-Through-Rate (CTR)* that captures user interactions like selecting, pausing, or seeking videos. This feedback helps adapt modalities' relevance and results in improved video representations.

### 3.2. Content-based recommenders

*Content-based recommenders*, also known as *Content-based Filtering (CBF)*, utilize item characteristics or features that users are interested in to find unseen items with similar attributes and present those as recommendations (Nikolakopoulos et al., 2022). The aim of CBF is to leverage the commonalities of item features that have been relevant to a *target user*, i.e., a user for whom a recommendation is computed, in the past, by suggesting items with high overlap in terms of similarity, determined by various similarity functions (Adomavicius and Tuzhilin, 2005).

Analyzing the publications on video recommenders revealed that content-based recommendations are predominantly computed using *supervised, unsupervised*, and *self-supervised* learning approaches. Table 3 classifies publication by these approaches. While supervised approaches determine whether an item is relevant or irrelevant to the target user, unsupervised approaches seek the most similar content based on the distance to a *seed* in the embedding space, where the seed describes the current user preference. Self-supervised techniques predominantly involve *Deep Learning* models to learn content structures for predicting item relevance.

**Table 3:** Content-based VRS approaches classified by applied technique and algorithms.

**Type**	**References**
Supervised learning	Luo et al., 2008; Zhu et al., 2013; Chantanurak et al., 2016; Elahi et al., 2017, 2020, 2021; Lee and Abu-El-Haija, 2017; Deldjoo et al., 2018a; Tavakoli et al., 2020; Hazrati and Elahi, 2021; Rimaz et al., 2021; Leite et al., 2022
Unsupervised learning	Wu et al., 2008; Davidson et al., 2010; Sanchez et al., 2012; Niu et al., 2013; Deldjoo et al., 2016, 2018b; Ramezani and Yaghmaee, 2016; Lu et al., 2017
Self-supervised learning	Mei et al., 2007, 2011; Covington et al., 2016; Gomez-Uribe and Hunt, 2016; Chen et al., 2018; Kaklauskas et al., 2018; Li et al., 2019; Jiang et al., 2020; Chakder et al., 2022; Du et al., 2022; Gong et al., 2022; Pingali et al., 2022; Yi et al., 2022; Mondal et al., 2023; Xiao et al., 2023

In the following, the publications and technical approaches to computing content-based recommendations are discussed in detail.

#### 3.2.1. Supervised learning

Supervised learning algorithms for content-based recommendation take the feature descriptions of items and user preferences (often defined as *user profiles*) as input to predict whether an item is relevant with respect to individual preferences. It comprises *classification*, i.e., the assignment of items to predefined categories like relevant/irrelevant, and *regression* analysis, i.e., the prediction of a numerical value like a user rating. Thereby, different features, feature representations, encoding of user preferences, and classification techniques are applied, depending on the context.

A predominantly used algorithm in content-based video recommendation is *k-Nearest-Neighbors (kNN)* (Luo et al., 2008; Zhu et al., 2013; Elahi et al., 2017, 2020, 2021; Lee and Abu-El-Haija, 2017; Deldjoo et al., 2018a; Hazrati and Elahi, 2021), which identifies the *k* most similar items, given a distance metric applied to the item features (Jannach et al., 2011b). Items are more similar, the lower the distance between them. In Chantanurak et al. (2016), this approach was used to recommend learning videos from YOUTUBE. It uses keywords from course metadata in a *Learning Management System (LMS)* as search queries to obtain a video selection and the available video keywords. Those are transformed to a TF-IDF representation, used for the kNN recommendation.

Besides comparing the similarity between video items, often a user profile reflecting the individual user preferences is used to identify similar videos. Mostly, this profile is based on past video consumption and represented in the same embedding space as the videos, which enables computation of the distance between them. An elaborated example is presented in Zhu et al. (2013), using a two-tower approach for the recommendation. In the video representation stage, a topic model based on textual and visual features is learned to describe the video. In the second stage, the user is described as a topic model based on their watch history. Relevant videos are identified by the minimal distance between the user model and topic models of videos.

Another supervised approach for content-based video recommendation is *Random Forest* (Ho, 1995). This machine-learning approach combines multiple decision trees to classify an item as relevant or irrelevant. The final decision is made through a majority vote. An example is presented in Tavakoli et al. (2020), where a model determining the relevance of learning videos to a user based on their current knowledge level and job skill requirements is developed, aiming to assess if a video matches a skill description in the educational video recommender.

#### 3.2.2. Unsupervised learning

Unsupervised learning algorithms for content-based recommenders extract patterns and relationships from unlabeled data to provide meaningful insights and recommendations without predefined categories. Clustering is one approach, which groups items such that items assigned to the same group (cluster) are more similar compared to others. For content-based recommendation, this approach is used to identify similar items to a seed or user preferences represented in the same embedding space. Any kind of content representation can be taken into account (see Section 3.1), and the approach is applicable to a variety of domains, e.g., for clustering sports videos based on recognized human actions (Ramezani and Yaghmaee, 2016) or using the identified topic of videos (Wu et al., 2008).

A popular clustering approach for video recommendation is *k-Means* (Wu et al., 2008; Deldjoo et al., 2016, 2018b; Ramezani and Yaghmaee, 2016), which is an iterative algorithm that assigns items to one of *k* clusters, such that the distance between the centroid (cluster center) and the item is minimized, given a distance metric (Jannach et al., 2011a). For a standard recommendation approach, clustering can be involved to identify the most similar cluster based on a user's context and recommend videos from that cluster that the user has not seen yet. Furthermore, clustering can also be beneficial in a two-stage recommendation process, where it helps generate an initial set of candidates from a large video catalog (Davidson et al., 2010). By using a fast clustering algorithm, the overall performance can be improved by prefiltering the videos, which are then ranked using a more accurate but slower algorithm. By taking neighboring clusters into account, the exploration of additional topics is favored, which can further improve the user experience (Wu et al., 2008).

Users may have distinct individual reasons for being interested in a video. For instance, one user appreciates the plot, while others are interested in the actors. In Lu et al. (2017), these factors are considered. Videos are clustered using a multinomial vector representation, where different topics are assigned to the same video with corresponding weights. Users are also modeled in this space based on their watch history, enabling the identification of the nearest cluster and recommending videos from that cluster.

*Spectral clustering* is an algorithm from the graph theory using eigenvalues of a similarity matrix to group items (Ng et al., 2001). In Niu et al. (2013), it is used to recommend videos based on the user's mood. The videos in this approach are clustered by their *affective* properties (see Section 3.6.1).

Another unsupervised approach is the usage of a *Hidden Markov Model (HMM)* (Baum and Petrie, 1966). In Sanchez et al. (2012), this has been used to recommend Olympic Games transmissions given a user profile and manually created video annotations. The system builds a user profile modeling user interests with weighted factors for preferences such as preferred sports and athletes. The profile evolves continuously based on consumed content using an HMM capturing the interest in specific videos as *hidden states*. The HMM parameters are used in a Bayesian inference step, to calculate the probability of video relevance to the user.

Also, *association rule mining* (Liu et al., 1998), which is a data mining technique that discovers relationships and patterns within large datasets based on item co-occurrence, can be used for content-based video recommendation. In Davidson et al. (2010), the approach is used to calculate a *relatedness score* of other videos in the catalog given a video watched by the user. This score represents the relations between videos as a directed weighted graph. A candidate set of items is then generated considering a limited transitive closure within a specified distance. The candidates are subsequently ranked based on various properties such as video quality (e.g., recentness and general popularity), user specificity (compatibility with the user watch history), and diversification (removal of similar videos to promote serendipity).

#### 3.2.3. Self-supervised learning

Self-supervised learning algorithms for content-based recommenders use automatically generated item embeddings (Chen et al., 2022b,c) as input to predict recommendations without requiring explicit user-item interactions. Those systems apply different types of neural networks to predict user ratings for videos using a variety of inputs.

In Kaklauskas et al. (2018), personal user characteristics are combined with real estate advertising videos in a *neuro decision matrix*, which is a cognitive framework employing neural network models to analyze complex patterns and data inputs enabling personalized decision-making. It is used to deliver personalized video clips showcasing properties matching individual preferences.

Pooling the video embeddings of positively rated videos using the feature-wise mean to obtain a user embedding is applied in Pingali et al. (2022). These user embeddings and embeddings of unseen videos are fed into a *Siamese neural network*, which is a neural network capable of comparing the similarity between two patterns. By utilizing a regression function, the method predicts ratings for similar videos. Using a *Graph Attention Network* (Chakder et al., 2022) or *Graph Convolutional Neural Network* (Mondal et al., 2023) to develop the regression system and extend movie embeddings with further latent features, the accuracy of this approach can be improved.

In Chen et al. (2018), a deep network-based method for the prediction of user clicks on micro videos is presented. The *Temporal Hierarchical Attention at Category- and Item-Level (THACIL)* network uses a combination of temporal windows to capture short-term dynamics of user interest, and multi-grained attention mechanisms to describe the diverse user interest. While category-level attention describes the diverse interest of users, fine-grained user interests are described with item-level attention. Using a hierarchical attention mechanism, short-term and long-term properties of user behavior are modeled.

Micro video recommendation faces the challenge of dynamic and diverse user interests, leading to the development of various solutions. One baseline strategy uses time decay to reduce the significance of videos watched further back in the past. An advanced version employs a temporal graph-guided network, as described in Li et al. (2019), to predict the click probability of videos. This model combines past user behavior with diverse topic preferences, considering both engaging and uninteresting videos from the user's viewing history. Furthermore, the model incorporates the notion of varying interest levels in topics, where actions such as liking a video are given higher importance than merely watching it.

Using a static time decay heuristic fails to consider personalized and individual preferences, where older videos might be more important for some users. In Jiang et al. (2020), a *Multi-scale Time-aware user Interest modeling Network (MTIN)* is proposed to address this issue. MTIN incorporates a parallel temporal mask network to capture varying importance over time. Additionally, the model utilizes a grouping approach for videos and assigns users to multiple interest groups, allowing for a more accurate representation of their diverse preferences.

To handle the dynamically changing user interests in micro-video applications, a real-time re-ranking solution was proposed in Gong et al. (2022). Recognizing that traditional server-side models might not capture short-term preferences from user interactions with minimal delay, the approach suggests deploying a lightweight edge-side model on the client side to re-rank the recommendations after each user interaction. This approach divides roles, utilizing server-side models for complex, enduring preferences, and enabling client-side models to incorporate immediate feedback for real-time adjustments.

CTR prediction, i.e., the anticipation of the following user action, is a challenge in video recommendation (Liu et al., 2020). In this context, the goal is to foresee a user's upcoming video choice based on their past interactions. Deep learning models based on the *Embedding and Multilayer Perceptron (MLP)* paradigm are commonly used for this task. These models map input features to low-dimensional embedding vectors, which are then transformed and concatenated in MLP layers to capture non-linear relationships among the features (Zhou et al., 2018). Nonetheless, this approach struggles with diverse user interests. For instance, if a user watches action, romantic, and science-fiction movies, merging all genres into a single representation might overlook genre-specific relevance due to the user's varied history.

To address this, the concept of *Deep Interest Networks (DIN)* was introduced in Zhou et al. (2018). DIN acknowledges that a portion of a user's interests can impact their subsequent actions, like choosing a movie. It dynamically computes the interest by considering historically significant actions related to a candidate item. A local activation unit with soft search identifies relevant portions of user history. A weighted sum pooling method generates an interest representation for the candidate item, assigning greater weights to more relevant segments. To incorporate user feedback into predictions using DIN, the *Preference Matching Network (PMN)* model was presented in Liu et al. (2020), following the idea that users are more inclined to accept candidate items that resemble videos they have positively rated. PMN first calculates similarity weights between a candidate video and the user's interaction history. Then, a weighted sum pooling of the user's feedback is calculated to determine their preference for the given candidate.

The exploration of user interest for CTR prediction as an extension to relying exclusively on historical behavior was suggested in Chen et al. (2022a). By explicitly modeling item relations and including them in the network for embedding user interest, recommendation quality can be improved.

In Xiao et al. (2023), a solution to tackle the cold start problem for new users was presented. The solution incorporates information from similar users in the social network. If the video platform shares users with a social network, a social graph can be created, capturing relationships such as friendships or common interest groups. Through clustering, similar groups of users can be identified. By aggregating the interests of these social groups with user features, the accuracy of personalized recommendations can be enhanced.

### 3.3. Collaborative filtering

Collaborative Filtering (CF) is based on the concept that users with similar preferences in the past will continue to have similar preferences. Hence, CF exploits past ratings to suggest unseen items by considering items liked by users with similar preferences (Ricci et al., 2015). The core assumption is that similar users share interests in similar items, and analogous items are favored by similar users (Nikolakopoulos et al., 2022). This involves identifying similar users, often termed as *neighbors*, by calculating the similarity of past ratings using measures like *Pearson correlation, cosine similarity*, or *Spearman's rank correlation coefficient* (Jannach et al., 2011a). Ratings can be explicit (direct user ratings or subscriptions) or implicit (derived from user behavior like viewing time) (Davidson et al., 2010; Koren et al., 2022).

For video recommendation, CF provides an intuitive approach, recommending unseen videos based on the preferences of users with similar interests. Table 4 groups various systems using this approach by their techniques. The summary shows that similar to the content-based recommendation (see Table 3), supervised, unsupervised, and self-supervised learning methods are widely used to compute recommendations.

**Table 4:** Collaborative filtering VRS approaches classified by applied algorithms.

**Type**	**References**
Supervised learning	Arapakis et al., 2009; Dias et al., 2013; Choi et al., 2016; Okubo and Tamura, 2019
Unsupervised learning	Wang et al., 2014; Ferracani et al., 2015; Katarya and Verma, 2016; Katarya, 2018; Tohidi and Dadkhah, 2020
Self-supervised learning	Hongliang and Xiaona, 2015; He et al., 2017; Rybakov et al., 2018; Yan et al., 2019
Further approaches	Baluja et al., 2008; Koren et al., 2009; Chen et al., 2015, 2019

In the following, the publications and algorithmic approaches of applying CF for video recommendation are discussed in detail.

#### 3.3.1. Supervised learning

Supervised learning in content-based and collaborative filtering diverges mainly in their used input. While CBF employs item content features to find similar items, CF operates on a user-item rating matrix along with the target user. CF utilizes nearest neighbors algorithms on the matrix to identify users who are similar to the target user. The process typically involves three steps (Dias et al., 2013): (1) Similarities between the target user and others are computed using ratings and a similarity metric. (2) The most similar users, known as neighbors, are selected. (3) Item ratings are predicted from the weighted average of neighbor ratings. While explicit ratings for the video or segments of a video (Dias et al., 2013) are frequently used, recommendations can as well be based on implicit ratings, for example, by applying emotion recognition to derive user preferences (Arapakis et al., 2009; Choi et al., 2016; Okubo and Tamura, 2019).

#### 3.3.2. Unsupervised learning

Video-based collaborative filtering often starts with clustering to decrease the search space of the model-based approach. Optimization methods are then used on similar user clusters, rather than the entire user space, to enhance scalability. Given the target user, the nearest cluster is identified, and video ratings are predicted using a weighted average of other users in the cluster.

Many methods use the k-Means algorithm for clustering similar users and enhancing the accuracy with varied optimization techniques. For instance, in Katarya and Verma (2016) *Particle Swarm Optimization (PSO)* is applied for improved cluster centroid assignment. The *Artificial Bee Colony (ABC)* algorithm optimizes user-cluster assignments (Katarya, 2018). In Wang et al. (2014), k-Means is paired with genetic algorithms in a two-step approach. Firstly, *Principal Component Analysis (PCA)* condenses data dimensions by removing less significant data. Secondly, this dense data is clustered to identify similar users.

Furthermore, the clustering itself can be improved. In Tohidi and Dadkhah (2020) evolutionary algorithms based on k-Means were used for this purpose. Alternatively, the *Fuzzy C-means (FCM)* algorithm, permits users to belong to multiple clusters with varying degrees of membership (Ferracani et al., 2015; Katarya, 2018). FCM optimally assigns users to these clusters, promoting a diverse user profile representation.

#### 3.3.3. Self-supervised learning

Self-supervised learning in collaborative filtering generates user vector representations reflecting their interests. Embeddings of users are compared using a distance metric to find target user neighbors. The weighted average of the neighbor's ratings is used to predict the item ratings used as recommendations.

In Hongliang and Xiaona (2015), a *Deep Belief Network (DBN)* quickly extracts user features, e.g., preferred genres and movie ages. User ratings are encoded as a binary matrix, where each movie corresponds to a column, and each rating value option is represented by a row (1 for rated, 0 for unrated). This matrix is then used as input for the DBN to generate a user feature vector. The feature vectors for all users are used to find nearest neighbors using the *Euclidean distance*.

Without explicit ratings, user preferences can be inferred from interactions as implicit feedback. The *Neural network-based Collaborative Filtering (NCF)* presented in He et al. (2017), takes the user and item ids as input features, converting them to binarized sparse vector with one-hot encoding. In the embedding layer, the item vector is projected to a dense representation, which is then fed into the multi-layer network for a prediction score. This score, obtained from the final layer, gauges video relevance for the target user.

In Rybakov et al. (2018), a two-layer neural network is trained to predict users' upcoming video selection. The model is designed to forecast videos to be consumed within a specific time frame, such as the upcoming week, leveraging the insight that predicting the next item is more accurate than random future items (Covington et al., 2016). This approach effectively captures both short-term trends, such as current events like the COVID-19 pandemic, and long-term user preferences. The model combines a *predictor* for currently popular items and an *auto-encoder* for static user preferences in a feed-forward neural network. The system is retrained daily to adapt to changes. The recommendation precision is improved by considering consumption dates through time decay, approximated through a convolutional layer.

As sparse user ratings can negatively impact the recommendation quality, the usage of sentiment analysis on free-text reviews is suggested in Mahadevan and Arock (2017) to address this issue. NLP techniques are used to deduce numerical ratings from credible reviews, which are then used in the recommendation process. Experiments showed improvements compared to the direct usage of ratings from the datasets. This highlights the potential of mapping text reviews to ratings for more meaningful user interest understanding than numeric ratings alone.

In video recommenders, personalized suggestions are typically based on user data like viewing history. However, in cold-start situations, where data is scarce, sharing information with other platforms or social networks can enhance user profiles. In Deng et al. (2013), two strategies were evaluated: (1) directly incorporating user profiles from an auxiliary platform to enrich the target platform, and (2) transferring user relationships (i.e., behavioral similarity) from the auxiliary to the target platform. This information was combined with user interactions on the video platform to compute personalized recommendations. Experiments revealed certain aspects of auxiliary profiles, such as shared articles and registration info, were more valuable than others. While integrating all data did not always improve accuracy and sometimes performed worse than relying solely on the target platform's sparse profile, selectively integrating relevant information from the auxiliary platform showed potential for performance improvement.

The discrepancy of user interests in different services, stating that user interest features include cross-site commonalities and site peculiarities, is observed in Yan et al. (2019). The study revealed, that *multi-homed users*, i.e., users using multiple services, have inconsistent and independent preferences in different services. Analogously, *multi-homed videos*, i.e., videos uploaded to multiple services, enable sharing of user interests across services. To tackle this, the study employs the *Deep Attentive Probabilistic Factorization (DeepAPF)* model, which splits user embeddings into common and site-specific parts, adapting feature weights via an attention mechanism. This approach captures both shared and unique user preferences across services.

In the domain of e-learning, cross-correlation of videos can be applied to leverage the use of videos across different courses, emphasizing the correlation of knowledge between courses (Zhu et al., 2018). This is achieved through a two-step approach: (1) CF is used to form a seed set of pertinent videos based on learner interactions like video view duration and navigation. (2) The degree of relevance between videos is computed using a cross-curriculum knowledge map, and a random walk algorithm is employed to measure the degree of relevance. This generates video subgraphs that contain video recommendations aligned with both learner preferences and the knowledge relevance of the video content.

#### 3.3.4. Further approaches

*Adsorption* is a graph-based semi-supervised learning approach that leverages user-video preferences for video recommendation (Baluja et al., 2008). It propagates known user preferences (labeled nodes) to unknown preferences (unlabeled nodes) based on the view history of users. Users and videos are represented as nodes in the graph, which are linked if users viewed them. Videos for recommendation are determined by identifying videos connected by short paths through other users.

*Singular Value Decomposition (SVD++)*, forms a powerful method for collaborative filtering that improves traditional matrix factorization (Koren et al., 2009). It includes implicit feedback and explicit user/item biases. The technique factors the user-item rating matrix into lower-dimensional matrices representing latent factors. These factors capture underlying features. The model approximates the original ratings by multiplying these matrices. To consider implicit feedback, a weighted regularization term is introduced, which considers the confidence of observed user-item interactions. This prioritizes highly relevant data. Explicit user/item biases handle inherent rating data biases, capturing individual user tendencies and item popularity.

In Chen et al. (2015), an *Artificial Immune System (AIS)* for CF is introduced. AIS mimics biological immune systems, comprising *antigens* (unclassified training data) and *antibodies* (generated in response to antigens). These antibodies construct specialized *immune networks* signifying their similarity to antigens, representing specific training data. After training, the final immune network predicts user ratings for a target user (antigen). This involves identifying nearest neighbors via similarity assessment of user groups (immune networks) and users within those groups (antibodies). By leveraging this immune system-inspired approach, accurate predictions can be made for the target user's ratings.

To handle the problem of unavailable explicit ratings, *Interest Preferences of Categories (IPoC)* can be deduced as implicit ratings from user logs (Chen et al., 2019). View times of short videos are used to determine ratings, reflecting user interest in specific categories through weighted video consumption times. These ratings are then used to fill a rating matrix for CF using matrix factorization. By weighing values higher for frequently consumed categories and factoring IPoC confidence, rating accuracy is enhanced.

### 3.4. Hybrid recommenders

Hybrid recommendation approaches combine various strategies to overcome the limitations of single recommendation strategies (Nikolakopoulos et al., 2022). Various hybridization designs are commonly employed (Jannach et al., 2011c). Firstly, the *parallel* design involves implementing multiple systems independently and combining their recommendations. Secondly, the *pipelined* design merges different approaches by using the output of one system as input for the subsequent recommender. Lastly, the *monolithic* design integrates diverse input data, e.g., item features and user ratings, into a single model.

The fundamental principle of hybrid recommenders is the integration of multiple strategies, like content-based and collaborative filtering, to overcome the limitations of individual methods, and enhance the accuracy and diversity of video recommendations. Hybrid systems commonly tackle data sparsity, scalability, and cold-start problems. An overview of the technical approaches used in publications is shown in Table 5.

**Table 5:** Hybrid VRS approaches classified by applied algorithms.

**Type**	**References**
Matrix factorization	Cui et al., 2014; Roy and Guntuku, 2016; Kvifte et al., 2021; Wang et al., 2021
Deep neural networks	Wang et al., 2015; Gao et al., 2017; Wei et al., 2017; Liu et al., 2019a; Chen et al., 2021
Multi-task learning	Ma et al., 2019; Zhao et al., 2019; Tang et al., 2020; Zhuo et al., 2021; Song et al., 2023
Further approaches	Öztürk and Kesim Cicekli, 2011; Vizine Pereira and Hruschka, 2015; Abbas et al., 2017; Liu et al., 2019b; Kim et al., 2021

In the following, the publications and algorithmic approaches for hybrid video recommendations are discussed in detail.

#### 3.4.1. Matrix factorization

Matrix factorization is an embedding model used to predict user ratings for unrated items. A characteristic of matrix factorization is the transformation of users and items in the same vector space, where both are clustered based on the similarity of latent factors (hidden features).

One option is to represent social media users and videos in a common attribute space (Cui et al., 2014). This method involves enriching videos with social aspects, like demographic data of viewers, and user profiles with content information from watched and liked videos. Experiments detected the appropriate balance of content and social attributes, favoring social attributes. This monolithic design aligns users and videos in a single attribute space, focusing on similarity-based matches for recommendations. For sparse videos, content similarities share social attributes, and user relationships share content attributes. The design effectively handles cold start for both items and users by mapping them to videos with similar content and common user relationships.

The model described in Roy and Guntuku (2016) emphasizes users' emotional influences on video preferences. It enriches collaborative data with recognized emotions users experience while watching videos. By integrating emotions, the model gains latent factors capturing emotional user-video connections. These latent factors are then used in a factorization method for rating predictions.

To improve the accuracy of recommendations in the presence of cold start and sparse ratings different approaches were suggested. In Kvifte et al. (2021), the usage of aggregated content data (visual features and word frequency in subtitles) and user ratings to predict recommendations via matrix factorization was presented. In Wang et al. (2021), a two-tower model is proposed to improve cold starts. One tower learns user embeddings from watch history, while the second tower learns item representations from metadata (e.g., genres, actors, and synopsis) and movie cover art. An attention layer weighs features based on item importance. Matrix factorization approximates user preferences with embeddings.

#### 3.4.2. Deep neural networks

Hybrid video recommenders using deep neural networks often aim to enhance recommendation accuracy by incorporating content features and user ratings. *Collaborative Deep Learning (CDL)* unites deep representation learning for content and collaborative filtering for ratings (Wang et al., 2015). This allows for a two-way interaction between the input information. Content features improve CF predictions and video ratings support feature learning using a *stacked denoising autoencoder (SDAE)*, which is a deep learning model that learns a hierarchical representation of data by removing noise and reconstructing clean input. Using this model, CDL generates accurate rating predictions for user-video pairs.

In Wei et al. (2017), the cold start problem is tackled by integrating an SDAE into the CF model *timeSVD++*. This model considers user preferences, item features, and temporal rating dynamics. The process starts by extracting and processing movie plots for relevant words. A bag-of-words vector captures item similarity. These vectors train the SDAE to extract item content features. The trained features are the input for the CF model that predicts the ratings of items with few or no ratings based on similar items which are already sufficiently rated.

*Dynamic Recurrent Neural Networks (DRNN)* (Gao et al., 2017) fuse dynamic user interest with content details. The system merges video semantics (textual and visual description), user interest from history, and user relevance (collaborative aspect) for similar user discovery. It adapts for single or cross-network use, possibly incorporating social networks for improved accuracy. Videos are represented in a semantic space using multi-modal features, and a common interest space connects semantics and user interest. An RNN models dynamic user interest over time, using a ranking loss constraint in the final RNN state to consider user relevance. This model acts as an interest network, harmonizing these sources to understand dynamic user preferences and provide interpretable user-video recommendations.

Hybrid approaches have also been implemented for micro-video recommendation. In Liu et al. (2019a), a model predicting if users will finish and like a video subsequently is described. The prediction model is learned from user interaction and multi-modal item feature data. To enhance the accuracy of predictions, an ensemble method is employed, utilizing individually predicted ranks from multiple prediction models. Notably, each model takes into account different time frames of the user's interaction history, leading to a more comprehensive understanding of user preferences and behavior.

In Chen et al. (2021), a method to combine various user interest representations for micro-videos and movies is presented. This approach fuses different representations of user interest, including the overall user profile, item and category-level representations, and collaborative data using a DNN. The outcome is a unified representation synthesized from different preference sources.

#### 3.4.3. Multi-task learning

*Multitask learning (MTL)* is a machine learning approach that trains one model for multiple related tasks, boosting performance through shared representations (Tang et al., 2020). In video recommenders, objectives can be diverse and sometimes conflicting. In that sense, the same system can have engagement objectives like clicks and watch time, while also considering user satisfaction indicated by likes or ratings (Zhao et al., 2019). MTL can help to tackle this challenge.

A model for combining three optimization goals, namely the partial order between videos, CTR, and prediction of the sequentially clicked video, was presented in Zhuo et al. (2021). Using a behavior-aware graph convolution network, the system differentiates user behaviors to reflect the influence between users and videos. Behaviors (e.g., clicks, watch duration, and ratings) are mapped to scores, adjusting interaction weight based on strength, where higher scores resemble greater user interest. Those weightings are merged into the embedding space of users and items. The model objective of learning is to estimate the probability of the target user choosing each of the available videos.

In Zhao et al. (2019), the ranking phase of video recommendation was enhanced by incorporating the *Multi-gate Mixture-of-Experts (MMoE)* architecture for MTL. MMoE has a shared bottom layer and separate expert layers per objective. The expert layers learn task-specific data from inputs. Gating layers for each task incorporate expert and shared input. The expert layer output is fed into a task layer predicting binary objectives (e.g., clicks and likes) or regression tasks (e.g., watch time, and ratings). In Song et al. (2023), MMoE is adapted for playback prediction, based on user history, embeddings, and playback time.

Those systems might suffer from the implicit *selection bias*, where the interaction logs used for model training do not capture whether users clicked on a recommended video because it genuinely matched their preferences or because it was simply ranked higher, potentially causing more relevant videos in the catalog to be overlooked. To mitigate this bias, a *shallow tower* alongside MMoE was added in Zhao et al. (2019). This tower uses inputs contributing to the selection bias (e.g., video position and device data) and integrates its output into the main model's final logit. This reduces bias and improves fairness and system efficacy.

*Progressive Layered Extraction (PLE)*, presented in Tang et al. (2020), forms an MTL approach improving shared learning efficiency while reducing *negative transfer* and the *seesaw phenomenon*. Negative transfer in RS occurs when unrelated objectives lower performance compared to single-task systems. The seesaw phenomenon is the trade-off between improved performance for one task and a decline in others in MTL. PLE is built on the *Customized Gate Control (CGC)* model, segregating shared and task-specific experts to avoid parameter interference. Task-specific experts focus on learning distinct knowledge, receiving input from their expert network and the shared expert network through a gating network for dynamic fusion. PLE extends CGC to a generalized model with multi-level gating networks and progressive separation routing, stacking CGC expert networks and creating extraction networks. Each extraction network receives fused outputs from lower-level networks, gradually learning deeper semantic representations and extracting higher-level shared information. By separating task parameters in upper layers, PLE enables the extraction of deeper semantic representations for each task, fostering generalization.

#### 3.4.4. Further approaches

A combination of the CF graph algorithm *Adsorption* with content-based similarity to improve the quality of recommendation was presented in Öztürk and Kesim Cicekli (2011). The system constructs a user-item graph, with users and items as nodes and weighted edges indicating interactions (e.g., likes). Items are initially labeled as relevant or unknown for each user. Adsorption spreads labels from labeled items to nearby ones, indicating relevance. Unrated videos reached via the graph are recommended. To improve the recommendations, the CF results are refined by including videos with similar content features, replacing less relevant suggestions.

Combining CF with *Demographic Filtering (DF)* (user profile creation from demographic characteristics) offers one possibility to address the cold start problem (Vizine Pereira and Hruschka, 2015). The *Simultaneous Co-Clustering and Learning (SCOAL)* algorithm uses video and user characteristics to create prediction models for different co-clusters, aiding users with minimal ratings by assigning them to the closest cluster. For users without any ratings, the cluster description and demographics determine the best prediction models. The first approach estimates the probability distribution for each co-cluster and calculates the predicted rating as a weighted sum, while the second, more resource-intensive method, constructs a video-by-video classifier involving only users who have rated the video.

The problem of sparse user ratings is addressed in Liu et al. (2019b) by computing user-video similarities using collaborative user similarity from ratings and content representation, which includes genre similarity and word embeddings from textual descriptions. These two similarities are fused using an adjusted weighted sum, which considers varying rating data importance. Ultimately, kNN recommends most similar videos based on these fused similarities.

### 3.5. Group recommenders

Group recommendation involves recommending items to a collective group rather than individual users, assuming the preferences of group members are known or can be obtained through recommender systems (Felfernig et al., 2018; Masthoff and Delić, 2022). Aggregating individual user models becomes a challenge in this approach, adding complexity to the recommendation process. An example of group recommendation is recommending a TV program that satisfies all viewers in a family watching TV together (De Pessemier et al., 2016).

In group video recommendations, the aim is to unite diverse individual user models with different strategies (Masthoff and Delić, 2022). For instance, in interactive television, the selection of programs should take into account the satisfaction of the entire group, not just the preferences of a single individual. Group recommenders face the particular challenge of balancing individual member satisfaction while suggesting items that align with the overall group preferences.

The *PolyLens* system(O'Connor et al., 2001), an extension of *MovieLens* (Harper and Konstan, 2015), focused on group movie recommendations. Users could create groups and receive movie suggestions based on collective group preferences rather than individual ones. Guided by a social value function, the process aimed to maximize the overall happiness of the group, gauged as the minimum happiness score among members. Recommendations excluded movies already viewed by some group members. Group suggestions were created by merging individual users' recommendation lists and ranking them based on least misery or decreasing social value. This method proved effective for smaller groups (2-4 people) with participants perceiving the generated recommendations as valuable and agreeing on their usefulness.

As an alternative to merging recommendation lists, the aggregation of user profiles to generate recommendations was presented in Yu et al. (2006). This technique is geared toward suggesting TV programs for groups watching TV together. The merging process combines vectorized feature descriptions of all group members' profiles by minimizing the total distance between them, aiming to retain the most common characteristics. To adjust for individual preferences, weight normalization is applied to the merged profile vector. By merging profiles and considering the collective characteristics, the system creates tailored recommendations for an enhanced TV experience.

The recommendation of movies for on-demand cinemas presents a unique application of context-aware group recommendation systems (Xue et al., 2019). This application focuses on combining classic cinemas with on-demand streaming, allowing groups to select movies in cinema rooms with specific equipment. Recommendations are essential for aiding guest decisions, though personalization is challenging due to the unknown and anonymous audience. The system addresses this by leveraging contextualization, considering temporal and spatial characteristics. Attendees are assumed to be local, and movie preferences vary based on the temporal aspect. Each cinema is expected to have its unique characteristics influenced by its environment captured by *Points of Interest (POI)* nearby. By collecting cinema activities like selected movies, time, and location, individual cinema profiles are created, integrating POIs, movie details, and ratings. Using this data, the system employs CF to model temporal and spatial dynamics. Temporal dynamics cover the *Periodic Effect* (common viewing patterns by time, day, and season), *Recency Effect* (preference for new movies), and *Audience Crowd Drifting Effect* (varying composition of audiences by time, such as couples or families). The spatial context is modeled through the Spatial Neighboring Effect (similar audiences in cinemas with similar POI patterns) and the *Spatial Popularity Effect* (differing regional movie popularity). This enables the prediction of movie ratings for specific cinemas at given times.

### 3.6. Further aspects

This chapter delves into various aspects of video recommenders, including the incorporation of affective signals like unconscious expressions and body language of users into RS, video recommendations tailored to consumption contexts, scenarios involving only certain parts of longer videos, publicly available datasets for VRS development, and an overview of metrics used to evaluate the recommendation quality.

#### 3.6.1. Affective computing

*Affective computing* aims to integrate human-like capabilities of perceiving, interpreting, and generating affect features, like emotions and mood in computers (Tao and Tan, 2005). This involves using sensors that capture diverse aspects of human behavior, such as gestures, voice, and heart rate, allowing computers to understand and respond in a friendly and intelligent manner. In recommender systems, this data enhances user profiles and feedback with unique information.

Using affective sensory data to automatically retrieve feedback is a popular method for determining user preferences in various video domains, such as TV program recommendation (De Pessemier et al., 2016), movies (Okubo and Tamura, 2019; Bandara et al., 2021), and advertisements (Choi et al., 2016; Kaklauskas et al., 2018; Kim et al., 2021). Facial expressions of users captured with webcams while watching videos provide more expressive opinions compared to simpler approaches, such as assuming that watching a video indicates liking (Arapakis et al., 2009; Choi et al., 2016; De Pessemier et al., 2016; Kaklauskas et al., 2018; Okubo and Tamura, 2019; Kim et al., 2021). Studies have shown positive correlations between identified smiles of users and video appreciation (Arapakis et al., 2009; Okubo and Tamura, 2019), but the correlation between emotions and ratings remains inconclusive in some cases (Diaz et al., 2018). Using DNNs, the emotion of users can be detected instantly to identify dynamic preferences and decide if recommended videos are appropriate (Choi et al., 2016; Kim et al., 2021). Since those approaches do not rely on a user history or a pre-existing profile, they offer a solution for cold-start situations in which the user is unknown.

In Kaklauskas et al. (2018), an affective VRS is designed to aid a variety of potential real estate buyers in discovering suitable properties. The system presents personalized property videos to users and records their facial expressions during viewing to gauge their emotional response. This data is utilized to determine whether to play another video clip and to identify the most suitable video from the catalog for the user.

Several VRS incorporate affective data for recommendations. In Roy and Guntuku (2016), the emotional connection between users and videos is modeled, suggesting users prefer videos they can emotionally connect with. To forecast emotional user reactions, a multi-label *Support Vector Machine (SVM)* classifier is used. SVM is a supervised machine learning method that determines an optimal decision boundary to classify data into classes, maximizing the margin between the closest data points of each class.

A related idea is applied in Niu et al. (2013) to recommend videos based on the user's current mood. The system utilizes a valence-arousal graph to autonomously learn affective attributes from videos. Valence signifies emotions from “pleasant” to “unpleasant,” while arousal measures the intensity of emotions from “excited” to “calm,” on a continuous scale. Recognizing that users' moods are dynamic and not static, the system captures users' affective traits within a session, encompassing sequentially watched videos. This approach assumes that the emotional impact of previously viewed videos influences the selection of the next video.

The usage of *Electroencephalograms (EEG)*, which measure brain neural activity, to capture user emotions and attention while watching videos is explored in Bandara et al. (2021). Using headbands, the brain activity of test users watching movie trailers was recorded. The EEG signals were classified into various emotional states, considering engagement and attention levels. Through EEG analysis, the system predicts video clip relevance to users based on their emotional and attention responses, which are then used for generating video recommendations.

In Leite et al. (2022), an affective virtual learning environment for algebra is examined. The system suggests learning videos according to the user's knowledge and engagement levels. It employs a sensor-free framework, using the user interaction log for predictions. Depending on both inputs, different categories of videos are considered for the recommendation. For instance, if a user's engagement is low and their knowledge is weak, the likelihood of recommending a video on a different topic is increased.

For an in-depth analysis of affective VRS, we refer to the comprehensive overview in Wang and Zhao (2022). The paper examines and categorizes the state-of-the-art in this field while identifying future research challenges. These challenges encompass the (1) scarcity of realistic high-quality datasets, (2) the integration of existing models with emerging deep-learning techniques, and (3) the adaptation of affective VRS for goals beyond accuracy, such as multi-task recommendations and explainable recommendations.

#### 3.6.2. Context-awareness

Context-aware recommender systems extend traditional recommenders by considering not only items and users but also the specific circumstances of the user when suggesting items (Colombo-Mendoza et al., 2015). These systems can be seen as a type of hybrid recommender, incorporating various factors to generate personalized recommendations. The context in this case refers to a combination of diverse attributes, including *spatial* context (location-related details) and *temporal* context (current time) and their impact on the recommendation process. Context awareness can be introduced to an existing video recommender by filtering or re-ranking its suggestions based on user context (Abbas et al., 2017). By tracking the user's context during video consumption, such as location or time, the system detects different contexts and then removes recommendations that do not align with the user's current context.

Addressing the challenge of identifying suitable contexts for videos watched by diverse users, the usage of *Soft-Rough sets* was proposed in Abbas and Amjad Alam (2019). While traditional *rough sets* handle incomplete or uncertain data by extracting patterns, they struggled to establish decision rules for video-context detection. Soft-rough sets, however, expand on rough sets by incorporating similarity degrees, enabling more flexible data classification and analysis. This extension helps in identifying the most fitting video context. In Abbas et al. (2019), a solution is introduced to address the problem of contextual sparsity in video recommendations, where relevant contexts are scarce due to insufficient data. Existing methods with uniform context weights often conflicted when choosing appropriate contexts for videos. To address this, a soft-rough set-based attribute reduction technique was employed. This technique identifies a minimal influential set of contextual factors that meet users' requirements within the VRS. Recommendations are drawn directly from computed soft sets of videos and contexts, with conflict-free recommendations being straightforward. In cases of conflict, attribute weights are determined by assessing the interdependency of contexts. Attributes that better differentiate contexts receive higher weights, aiding in selecting pertinent contexts for a given video set.

#### 3.6.3. Segments of interest

*Segments of Interest (SOI)* are video parts that users highlight while watching because they are interesting to them. The intention is that users like specific parts of videos more than others. In Dias et al. (2013), users with overlapping SOIs in different videos are assumed to have similar tastes and are selected as nearest neighbors for video recommendations. The SOI similarity is used to increase the similarity between users with overlaps proportionally, impacting the nearest neighbor computation while avoiding issues when no segments are highlighted yet.

An alternative approach to highlight SOI is introduced in Ferracani et al. (2015). Users annotate outstanding frames with comments and add semantic references to WIKIPEDIA.^12^ These annotations are used to cluster the video into a hierarchically structured taxonomy using the fuzzy k-Means algorithm. Videos are represented as vectors of weighted categories, used to determine video similarity. Relevance to users is assessed by merging implicit and explicit ratings.

#### 3.6.4. Datasets

Publicly available datasets are valuable resources for researchers to compare the results of offline experiments and enable reproducibility. This way benchmarks and leaderboards can be created, providing an overview of the state-of-the-art performance in specific domains. In the field of RS, platforms like *Papers With Code*^13^ offer benchmarks for various datasets, including those relevant to VRS, fostering accessibility to datasets with diverse characteristics.

One of the most used datasets for RS and especially VRS are the *MovieLens* datasets (Harper and Konstan, 2015). Launched by researchers at the University of Minnesota in 1997, *MovieLens* is a movie recommendation system that allows users to rate movies and receive personalized recommendations based on their ratings. Based on the collected data of this service, multiple versions of the dataset with different sizes have been released over the years, making it a standard benchmark for recommender algorithms in research and education.

The NETFLIX dataset (Bennett and Lanning, 2007), released in 2006 alongside the *Netflix prize* challenge, contains anonymous movie ratings by users. The challenge aimed to outperform the accuracy of the *Cinematch* baseline by 10%, measured using *Root mean squared error (RMSE)* as metric. The goal was to predict the number of stars a user would rate a movie on a 1 to 5 scale. This competition resulted in significant advancements in RS, with matrix factorization methods becoming key technologies for collaborative filtering, surpassing classical nearest-neighbor techniques. The winning solution is detailed in Koren (2009).

The Supplementary material of this paper offers a range of datasets for assessing and enhancing VRS. These datasets are outlined with a short description. Most datasets are suitable for content-based and collaborative filtering, with fewer incorporating context awareness and affective signals. Entertainment domains, particularly movies, dominate the dataset landscape, with fewer options for domains like e-learning, resulting in fewer research publications in those areas. This scarcity of specialized datasets emphasizes the need for more domain-specific datasets to foster research in various areas.

#### 3.6.5. Evaluation metrics

Evaluation metrics are essential in VRS experiments, offering insights into recommendation quality. Consistent metrics across publications enable system comparison and finding suitable approaches. A wide range of metrics assess various quality aspects, including accuracy, coverage, novelty, and scalability, across different item types, including videos. A comprehensive overview of RS evaluation, including offline and online settings, is available in Gunawardana et al. (2022).

In the context of video recommendations, *unexpectedness* was introduced as a unique concept in RS in Adamopoulos and Tuzhilin (2014). Unlike *novelty*, which suggests unfamiliar items, unexpectedness recommends items that deviate from user expectations but are still perceived as beneficial. *Serendipity* goes further, requiring user appreciation for the recommendation and excluding items that are not novel, while unexpectedness may include surprising but known items. *Diversification* enhances item variety through post-processing by removing or replacing similar items, unlike unexpectedness, which affects recommendation generation. Integrating unexpectedness with accuracy can enhance overall user satisfaction. In addition, the *Bayesian Surprise* measures computational creativity by quantifying surprise as the distance between user expectations, aiding the development of creative and surprising recommendations (Lu et al., 2018).

## 4. Discussion

In recent years, various approaches have been introduced for recommending videos in different situations. Due to the complexity and diversity of applications, there is no single solution that can be universally applied in all contexts. The choice of the appropriate approach depends on specific objectives. Addressing various challenges requires different mitigation strategies, which will be discussed in the following section, and finally, concluded by highlighting potential areas for future research and addressing unresolved issues.

Content-based video recommendation approaches do not rely on user communities and are applicable to individual users by understanding their interests and the available content. These methods suggest videos with content most similar to the user's preferences (Adomavicius and Tuzhilin, 2005; Jannach et al., 2011b; Nikolakopoulos et al., 2022). However, knowledge about user interests is crucial, which can be acquired explicitly through ratings (Lee and Abu-El-Haija, 2017) or direct preferences (Sanchez et al., 2012; Tavakoli et al., 2020), or implicitly through user-system interactions (Mei et al., 2007, 2011; Liu et al., 2020).

A more advanced method for automatically gathering implicit feedback involves the utilization of affective sensors, which is a popular topic of active research. These sensors have the potential to enhance the interpretation of implicit feedback, leading to improved recommendations (Choi et al., 2016; Kaklauskas et al., 2018; Okubo and Tamura, 2019; Kim et al., 2021). However, their widespread adoption faces uncertainty due to user acceptance and privacy concerns, particularly for more complex devices like EEGs (Bandara et al., 2021). Ensuring responsible usage and compliance with privacy laws, such as GDPR^14^, is crucial to building user trust in such technologies.

In general, content-based approaches have some common weaknesses (Adomavicius and Tuzhilin, 2005; Nikolakopoulos et al., 2022): (1) *Limited content analysis* arises from incomplete or insufficient information about items and users, hindering personalized recommendations. (2) *Over-specialization* occurs as these approaches mainly focus on suggesting similar items to those previously liked, potentially missing diverse content relevant to the user. (3) The *cold start problem* describes a ramp-up phase of new users to a system, requiring new users to provide enough ratings for the system to generate useful recommendations, which may take time.

To address challenges like the cold start problem and limited content analysis, automatic extraction of features has proven effective in representing video content for recommendation (Luo et al., 2008; Ramezani and Yaghmaee, 2016; Lee and Abu-El-Haija, 2017; Hazrati and Elahi, 2021; Rimaz et al., 2021). The selection of features impacts recommendation quality, with different multimedia features showing varying effectiveness across video domains. For instance, in domains rich in information density like education or news, textual features appear to provide the most valuable content description (Luo et al., 2008; Chantanurak et al., 2016; Kimoto et al., 2016; Tavakoli et al., 2020). In contrast, in entertainment domains, especially visual features appear to offer a good basis for calculation of recommendations (Deldjoo et al., 2016, 2018b; Lee and Abu-El-Haija, 2017; Elahi et al., 2020, 2021; Yi et al., 2022).

Combining multiple features of different types can improve recommendation quality in some cases (Elahi et al., 2017; Deldjoo et al., 2018a). However, this is not universally valid. For instance, combining stylistic visual features with textual content descriptions in the movie domain may reduce quality due to semantic dissimilarity (Deldjoo et al., 2018b). In some cases, using low-level visual features individually outperforms their combination due to the lack of correlation between aspects (Deldjoo et al., 2016). The quality of recommendations also depends on the aggregation strategies used (Mei et al., 2007, 2011; Chakder et al., 2022; Pingali et al., 2022; Mondal et al., 2023), with different contexts requiring different aggregation approaches for better performance.

In Section 3.2, various algorithms with distinct requirements for optimal performance were identified. Supervised learning techniques excel with good feature descriptors, particularly when leveraging textual features (Sanchez et al., 2012; Tavakoli et al., 2020). They work well even with limited user information, making them valuable for new users (Sanchez et al., 2012). Unsupervised techniques perform effectively with sparse feature descriptions, enabling the retrieval of meaningful topic descriptors (Wu et al., 2008; Lu et al., 2017). For entertainment videos, automatically extracted low-level visual features are well-suited for clustering-based recommendations, outperforming manually added textual features (Deldjoo et al., 2016, 2018b). Clustering also helps maintain performance in large item catalogs, as only the most similar clusters to the user profile need consideration. Self-supervised approaches are suitable for large catalogs, especially when used in conjunction with automatically extracted features. Deep neural networks are often applied for CTR prediction to recommend videos the user is likely to watch next (Covington et al., 2016; Liu et al., 2020). Multi-modal features are effective for video representation, capturing hidden commonalities between items and utilizing comprehensive descriptions for robust recommendations (Chakder et al., 2022; Pingali et al., 2022; Mondal et al., 2023).

With the availability of user ratings, collaborative filtering is a widely used technique for video recommendation, especially in scenarios with many users. Unlike content-based approaches, CF does not require content analysis, as long as explicit or implicit ratings are present (Jannach et al., 2011a; Nikolakopoulos et al., 2022). However, CF systems face two kinds of cold start problems: (1) the *new user problem* requires new users to provide enough ratings, and (2) the *new item problem*, where new items require enough ratings to be recommended. Furthermore, the *sparsity* of ratings challenge those systems, as a sufficient number is crucial for accurate recommendations (Adomavicius and Tuzhilin, 2005).

For collaborative filtering in video recommendation, the kNN method is frequently used. Similar users are identified as neighbors based on their rating patterns, and their ratings are used to predict ratings for the target user (Dias et al., 2013). To handle large user datasets and maintain sufficient performance, clustering is applied to focus on relevant data subsets (Katarya and Verma, 2016; Katarya, 2018). To address the sparsity of ratings, implicit feedback is employed to learn preferences from past user interactions (He et al., 2017; Rybakov et al., 2018). Especially self-supervised approaches have demonstrated effectiveness in handling implicit ratings efficiently.

In general, CF approaches are effective in avoiding overspecialization and enhancing recommendation quality in terms of serendipity, regardless of the specific method used. This was demonstrated with the winning system of the *Netflix prize*, which employed matrix factorization techniques (Koren et al., 2009).

To mitigate cold start situations for new users in CF, sharing user information across multiple platforms or social networks can be effective in providing initial user profiles (Deng et al., 2013; Yan et al., 2019). However, its real-life applicability is limited to cases where one provider offers multiple services and can share data between them, with privacy protection being a critical consideration. Alternatively, using demographic information for initial recommendations to new users can be helpful (Cui et al., 2014; Vizine Pereira and Hruschka, 2015), extending CF to a hybrid approach.

Hybrid video recommenders combine different methods to overcome individual limitations. To address cold start for new users, hybrids merge CF with CBF by enriching user profiles from other sources (Cui et al., 2014; Vizine Pereira and Hruschka, 2015) or augmenting items with content descriptions (Öztürk and Kesim Cicekli, 2011; Wang et al., 2015, 2021; Gao et al., 2017; Mahadevan and Arock, 2017; Wei et al., 2017; Liu et al., 2019b; Kvifte et al., 2021). The latter is particularly helpful in mitigating the sparsity of user ratings. Additionally, Multi-Task Learning can be used to effectively combine multiple objectives within a single VRS (Zhao et al., 2019; Tang et al., 2020).

By adding context information to video recommenders, the challenge of changing user interests based on spatial or temporal context can be addressed. These systems incorporate information about when and where users consume videos, allowing them to provide more relevant and useful recommendations, ultimately enhancing the overall user experience (Abbas and Amjad Alam, 2019; Abbas et al., 2019).

As a summary, we conclude our findings in Table 6 by outlining the advantages and disadvantages of the different approaches for video recommendation. While content-based methods serve as a good standard approach for video recommendation when at least basic feature descriptions exist or can be generated, the incorporation of user ratings enables the utilization of collaborative methods, which frequently enhance the generation of unexpected suggestions. However, these methods require a ramp-up phase to be able to suggest useful videos. A hybrid approach that merges content features with collaborative data presents a good opportunity to alleviate the limitations and leverage the advantages of each approach.

**Table 6:** Advantages and disadvantages of different recommendation approaches in the video domain.

**Content-based RS**		**Collaborative Filtering**		**Hybrid RS**	
**Advantages**	**Disadvantages**	**Advantages**	**Disadvantages**	**Advantages**	**Disadvantages**
No user community required	Modeling of content representation	No need for content representation	Sufficiently large user base required	Mitigate cold start for new users	Increased maintenance cost
High scalability	Learning user preferences	Serendipity	Cold start for new items	Mitigate low number of ratings	Computational complexity
No cold start for new items (extracted content features)	Cold start for new users	No explicit modeling of user preferences	Cold start for new users	Extension of user profiles with other sources	
Niche item recommendation	Overspecialization due to focus on similarity	Offline computation		Consideration of user context	

In cases, where the recommendation of videos is directed toward multiple persons instead of individuals, group recommender systems are able to suggest content that satisfies the preferences of multiple users simultaneously. The challenge is to balance diverse user profiles and recommend items in a suitable order (O'Connor et al., 2001; Yu et al., 2006). While group recommendation can be beneficial, it is not widely used for videos compared to individual user-based approaches. However, it offers potential advantages, such as more expressive ratings when different criteria are rated separately, to understand why a user likes the video, and compute recommendations based on those criteria (Felfernig et al., 2018; Masthoff and Delić, 2022). Furthermore, cold start situations can be mitigated by using social filtering to extend user profiles with information from similar users.

### 4.1. Research issues

Our literature overview on video recommender systems highlights several potential research directions for further exploration in this field. These directions will be elaborated on in the following.

#### 4.1.1. Bias and manipulation

Recent attention has been drawn to bias in video recommendations, particularly in social and political contexts, like elections and the COVID-19 pandemic. Platforms like YOUTUBE are accused to steer users in specific directions or causing filter bubbles, and spreading misinformation. Yet, publications analyzing bias in video recommendations are scarce. One such study (Kirdemir et al., 2021) investigated bias in YOUTUBE's algorithm, finding that a few videos are recommended noticeably more frequently, creating a bias toward popular videos. In Papadamou et al. (2022), the recommendation of pseudoscientific content, e.g., videos promoting conspiracy theories, on YOUTUBE was analyzed to observe the self-reinforcing effect of the view history, showing that countermeasures to fight misinformation are part of the recommendation algorithm.

Besides bias, manipulating recommendations is a significant concern explored across various item domains (Hurley, 2011; Adomavicius et al., 2013), particularly on social media platforms (Lang et al., 2010). The study in Edwards et al. (2022) illustrated a successful attack on a content-based recommender using manipulated videos, where subtle modifications to video visual features affected the model's content interpretation, while it was not recognizable to the human eye.

Based on this initial research, improving the understanding and increasing the awareness of bias in video recommendation can be a promising research area. Furthermore, researching methods for detecting and preventing manipulation also presents a potential for future work.

#### 4.1.2. Few-shot and zero-shot video recommendation

Recently, neural network models capable of *few-shot* and *zero-shot* classification, like, for example, *CLIP* (Radford et al., 2021), gained increasing attention. Those models are able to accurately predict labels with few (few-shot) or none (zero-shot) labeled examples. While these models already have been shown to outperform other approaches in interactive video retrieval (Lokoč et al., 2023), their potential in video recommendation remains largely unexplored. Future research could focus on applications in recommendation systems where historical interaction data is limited or absent, potentially improving cold start scenarios. Additionally, the possibility of developing generalized models capable of accurately recommending videos across diverse domains offers potential for future work.

#### 4.1.3. Live stream recommendation

Incorporating recommenders in live stream scenarios presents a promising field with real-time performance requirements. While real-time feedback analysis via affective sensors has been explored (see Section 3.6.1), limited attention has been given to live content analysis. For instance, in Dai et al. (2014), an approach using OCR and figure recognition on keyframes has been proposed to detect text and suggest related videos during live streams, like showing additional videos of a scoring football player. The key challenge involves rapid feature extraction and computation to understand live stream content for timely recommendations. A potential direction for future research could involve exploring various options for applying recommendations in live stream contexts.

#### 4.1.4. Knowledge-based video recommendation

Knowledge-based recommender systems leverage information about items and users to make reasoned decisions about which items align with user requirements in an interactive manner (Burke, 2000; Felfernig and Burke, 2008). Users specify their preferences, and the system attempts to identify suitable items. If none are found, user requirements might need adjustment (Jannach et al., 2011d). While this approach is well-established in various domains, particularly in cases where items are complex or users have limited knowledge about them, e.g., financial services, it remains underexplored for videos. This scarcity of publications might be related to the perceived high cost of defining recommendation knowledge for large video catalogs. However, in domains like learning videos, knowledge-based systems could be beneficial, allowing users to express their knowledge and refine their requirements iteratively, as outlined in Lubos et al. (2022). Users with general learning goals can outline their existing knowledge as requirements, allowing iterative refinement. Case-based systems (Jannach et al., 2011d), which allow users to refine their requirements iteratively, could guide users to appropriate videos. Initial studies in this area can be valuable to assess the applicability of knowledge-based approaches for video recommendations.

#### 4.1.5. Multi-modal content representation

Video items are characterized by multi-modality, incorporating various dimensions that describe their content (see Section 3.2). yielding rich information potential yet posing efficiency challenges in representation. While existing studies (Mei et al., 2007, 2011; Chakder et al., 2022; Pingali et al., 2022; Mondal et al., 2023) address this topic, many questions remain unanswered. Future research can focus on the analysis and development of methods to aggregate multi-modal features, across diverse video domains and applications, to determine effective strategies for specific scenarios. Furthermore, a performance comparison between recommenders using aggregated feature descriptions and systems aggregating the suggestions of multiple systems operating on distinct dimensions could be considered. This could help identify effective strategies for content representation and recommendation.

#### 4.1.6. Non-entertainment datasets

Most video recommendation datasets concentrate on the entertainment domain, particularly movies (see Section 3.6.4). This leaves a gap in publicly available datasets from other domains like e-learning, where the content is substantially different. As a result, evaluation outcomes derived from entertainment datasets might not accurately reflect system performance in other scenarios. Given the increasing significance of videos across diverse domains, particularly in knowledge transfer, there is a need for advancing research and introducing new datasets to aid the development of specialized systems.

#### 4.1.7. Scalability

As the demand for personalized video recommendations grows, video streaming companies face challenges related to hardware and network traffic. To ensure a stable service, cloud servers are distributed. However, this can lead to localized biases in recommendations based on user preferences in that area (Duan et al., 2020). For instance, if a local server serves mainly young users who prefer educational content, older users with different interests might receive inappropriate suggestions. Therefore, one potential for further research can be identified in the distribution of RS on cloud and edge infrastructures, facing the challenges of network load and performance to provide good results in general. The *JointRec* framework, presented in Duan et al. (2020), proposes the *JointCloud* architecture in mobile IoT, using distributed training across servers to mitigate biases and provide competitive results. Further research might explore the potential of distributed VRS in cloud and edge infrastructures.

#### 4.1.8. Segment recommendation

Current video recommender systems primarily focus on suggesting complete videos, which is well-suited for entertainment content. However, in domains like news or education, recommending specific video segments can be more advantageous, as users may only be interested in specific parts of the whole video (see Section 3.6.3). For instance, in knowledge transfer, suggesting relevant segments based on a user's existing knowledge can enhance efficiency by avoiding the repetition of known topics. Future research could explore methods to recognize feedback on specific video parts and interpret this feedback to identify segment borders. Additionally, incorporating user knowledge into their profile preferences is crucial for providing valuable recommendations in such scenarios.

## 5. Conclusion

This article offers a comprehensive overview of recommendation approaches in the video domain. The methodology used in this study analyzed recent publications, categorizing them based on their underlying recommendation approaches. By examining the various systems, we highlighted their respective strengths and weaknesses, providing valuable insights for selecting the most suitable approach for specific application contexts. In this overview, we identified the challenges and opportunities faced by video recommender systems. By improving the understanding of limitations and potential areas of improvement, we aim to inspire further research and development in the field.

## Author contributions

SL: Writing—original draft, Writing—review & editing. AF: Writing—review & editing. MT: Writing—review & editing.

## Footnotes

^1^ www.youtube.com

^2^ www.netflix.com

^3^ https://dl.acm.org

^4^ https://scholar.google.com

^5^ https://www.researchgate.net

^6^ https://www.sciencedirect.com

^7^ https://link.springer.com

^8^ https://www.imdb.com/

^9^ http://www.omdbapi.com/

^10^ https://lod-cloud.net

^11^ https://www.tiktok.com

^12^ https://www.wikipedia.org

^13^ https://paperswithcode.com/task/recommendation-systems

^14^ https://gdpr.eu
