# Supplementary material for: An overview of video recommender systems: state-of-the-art and research issues
Source: Front Big Data. 2023 Oct 30;6:1281614. doi: 10.3389/fdata.2023.1281614 (PMC10642507; doi:10.3389/fdata.2023.1281614)
Supplement: Supplementary file 1 [file Data_Sheet_1.pdf]

An Overview of Video Recommender Systems: State of the Art and Future Research Issues - Supplementary Material

1 DATASETS FOR VIDEO RECOMMENDER SYSTEMS

The table below shows a summary of different publicly available datasets for the evaluation and development of video recommender systems.

Table S1: Overview of publicly available datasets for the development and evaluation of video recommender systems. The *Content Type* describes for which type of data is included.

Name	Content Type	Description
Amazon Review[1] Ni et al. (2019)	Content description and user ratings	Updated version of the *Amazon Product* dataset, containing user reviews and ratings for different product categories, including around 8.7 million reviews of approximately 203000 products in the category ”Movies and TV”, collected between 1996 and 2018. Additionally, the descriptions of the products as presented on the detail page of the online shop are included as metadata.
Anime Recommendations[2] CooperUnion (2017)	Content description and user ratings	Dataset containing a basic description, including genre, title, type, of 12294 anime movies and series and their received ratings from 73516 users.
AudioLens[3] Rimaz et al. (2021)	Content description and user ratings	Extension to 9104 movies included in the MovieLens dataset, adding the reference to their soundtracks on Spotify and automatically extracted audio feature metadata, including acousticness, danceability, energy, instrumentalness, liveness, loudness, popularity, speechiness, tempo, track duration, valence, key, mode and time signature. The included movies form a subset of the MovieLens 25M dataset, with around 18.7 million ratings from 16.254 users.
DiDeMo[4] Hendricks et al. (2017)	Content description	Dataset containing over 10000 unedited, personal videos, with different visual settings. The videos are segmented and labeled with descriptions, so actions in segments can be distinguished.
MSR-VTT[5] Xu et al. (2016)	Content description	Dataset with 10000 web video clips, with a total duration of 41.2h, labeled with natural sentence descriptions (multiple labels per clip).
Movie Description Rohrbach et al. (2015)	Content description	Dataset with more than 54000 video snippets from 72 movies, labeled with movie script captions and audio descriptions (used for disabled persons).
IPTV[6] Sun and Yang (2021)	User ratings	User data collected from a Chinese IPTV service provider, representing the information of the start time and duration users watched each of the 164 channels. The device id is used to distinguish between the 221000 subscribers included in the dataset.

Continued on next page

Table S1 – continued from previous page

Name	Content Type	Description
LDOS-CoMoDa[7] Košir et al. (2011)	Context-based content description and user ratings	User ratings for various movies including contextual information capturing the situation in which the movies have been consumed in terms of 12 categorial values, including time, season location, etc. Additionally, demographic data and brief movie descriptions are provided.
MicroVideo-1.7M[8] Chen et al. (2018)	Content description and user ratings	Dataset with more than 12 million interactions of 10986 users made on around 1.7 million micro videos. For each video, visual features of the cover image are available, as well as a manually assigned category label (512 categories in total). Interactions are associated with user and video ids and include positive and negative interactions.
MovieLens[9] Harper and Konstan (2015)	User ratings	Collection of various datasets with different sizes and slightly different metadata. All provided datasets include user preferences as tuples of (user, item, rating, timestamp) indicating the user, rated item, rating, and time the item was rated. Basic content descriptions including genre, title, etc. are included and can be extended by linking them with the metadata of the *Internet Movie Database (IMDb)*[10], using the mapping of MovieLens ids to IMDb ids included id in the more recent datasets.
MMTF-14K Deldjoo et al. (2018)	Content description	Audio and visual feature descriptors of movie trailers describing the characteristics of 13623 movies. Additionally, textual metadata and user ratings are provided together with benchmark results for uni-modal and multi-modal recommender systems.
MovieTweetings[11] Dooms et al. (2013)	User ratings	More than 900000 ratings for 38018 movies of 71707 different users derived from well-structured tweets collected on Twitter. The dataset contains mappings of user ids to their Twitter ids, some movie metadata, including title and genre, as well as ratings and the time when the rating was provided. Ratings are given on a scale from 0 to 10.
Netflix Prize[12] Bennett and Lanning (2007)	User ratings	More than 100 million ratings from 480000 randomly chosen Netflix customers for beyond 17000 movies, collected between 1998 and 2005. Movies are rated on a 5-point scale and only include their title and year of release as descriptions.
TikTok[13] Jafarian and Park (2021)	Content description	Labeled short videos of single persons dancing as part of a dance challenge. The dataset includes extracted frames, where the person is segmented to enable the possibility to analyze the pose separately.

Continued on next page

Table S1 – continued from previous page

Name	Content Type	Description
Vevo Music Graph[14] Wu et al. (2019)	Content description	60740 music videos of 4435 artists of the Vevo music platform collected from YouTube. It includes collected metadata, e.g. title, description, tags, category, the view count time series collected during 63 days in 2018, and recommendations presented on YouTube for the videos in the given time.
Video Emotion[15] Roy and Guntuku (2016)	Affective content description	323 video clips, including 1917 ratings of 111 users. The videos are enriched by labels of 9 different emotions and an assigned valence score for those.
Yahoo! Movies[16] Labs (2016)	Content description and user ratings	Movie ratings and descriptions split in training and test dataset collected before November 2003. The training set contains 7642 users which rated 11915 different movies on a 5-point rating scale. The test set contains 2309 users which rated 2380 different movies. Additionally, it contains the content descriptions of the movies, including reviews, plot summaries, actors, directors, etc., as well as mapping to the MovieLens dataset. The links to posters, previews, and reviews are mostly not available anymore.
Yahoo! News Videos[17] Labs (2015)	Content description	21026 news videos (split in training and test set) covering various topics. The dataset contains descriptions of the video content in terms of categories, visual statistics, e.g. frames and shots, textual statistics, e.g. tokens and entities, and aural features, e.g. frequency and loudness. Only around one-third of the referenced videos are still accessible.
YouTube-8M[18] Abu-El-Haija et al. (2016)	Content description	Multi-label video classification dataset composed of around 6 million videos that are automatically annotated with 3800 visual entities using the YouTube video annotation system. It does not contain rating information. Additionally, a subset of the videos was collected in a segment dataset with human-verified labels, at which point of the video the labels occur to enrich it with time information.

[1] https://nijianmo.github.io/amazon/index.html

[2] https://www.kaggle.com/CooperUnion/anime-recommendations-database

[3] https://github.com/mhrimaz/audio-lens

[4] https://github.com/LisaAnne/LocalizingMoments

[5] https://www.microsoft.com/en-us/research/publication/msr-vtt-a-large-video-description-dataset-for-bridging-video-and-language

[6] https://dx.doi.org/10.21227/wqy2-h486

[7] https://www.lucami.org/en/research/ldos-comoda-dataset

[8] https://github.com/whn09/THACIL

[9] https://grouplens.org/datasets/movielens

[10] https://www.imdb.com/

[11] https://github.com/sidooms/MovieTweetings

[12] https://www.kaggle.com/netflix-inc/netflix-prize-data

[13] https://www.yasamin.page/hdnet$_tiktok$

[14] https://github.com/avalanchesiqi/networked-popularity

[15] Available upon request

[16] https://webscope.sandbox.yahoo.com/catalog.php?datatype=r

[17] https://webscope.sandbox.yahoo.com/catalog.php?datatype=r

[18] https://research.google.com/youtube8m/index.html

REFERENCES

Abu-El-Haija, S., Kothari, N., Lee, J., Natsev, P., Toderici, G., Varadarajan, B., et al. (2016). Youtube-8m: A large-scale video classification benchmark. *CoRR* abs/1609.08675

Bennett, J. and Lanning, S. (2007). The netflix prize

Chen, X., Liu, D., Zha, Z.-J., Zhou, W., Xiong, Z., and Li, Y. (2018). Temporal hierarchical attention at category- and item-level for micro-video click-through prediction. In *Proceedings of the 26th ACM International Conference on Multimedia* (New York, NY, USA: Association for Computing Machinery), MM '18, 1146–1153. doi:10.1145/3240508.3240617

[Dataset] CooperUnion (2017). Anime recommendations database

Deldjoo, Y., Constantin, M. G., Schedl, M., Ionescu, B., and Cremonesi, P. (2018). Mmtf-14k: A multifaceted movie trailer feature dataset for recommendation and retrieval. In *Proceedings of the 9th ACM Multimedia Systems Conference, MMSys2018, Amsterdam, The Netherlands, June 12-15, 2018* (ACM), 450–455

Dooms, S., De Pessemier, T., and Martens, L. (2013). Movietweetings: a movie rating dataset collected from twitter. In *Workshop on Crowdsourcing and Human Computation for Recommender Systems, CrowdRec at RecSys 2013*

Harper, F. M. and Konstan, J. A. (2015). The movielens datasets: History and context. *ACM Trans. Interact. Intell. Syst.* 5. doi:10.1145/2827872

[Dataset] Hendricks, L. A., Wang, O., Shechtman, E., Sivic, J., Darrell, T., and Russell, B. (2017). Localizing moments in video with natural language

Jafarian, Y. and Park, H. S. (2021). Learning high fidelity depths of dressed humans by watching social media dance videos. *CoRR* abs/2103.03319

Košir, A., Odic, A., Kunaver, M., Tkalcic, M., and Tasic, J. F. (2011). Database for contextual personalization. *Elektrotehniški vestnik* 78, 270–274

[Dataset] Labs, W. . Y. (2015). R11 - yahoo news video dataset, version 1.0. Accessed 11 May 2022

[Dataset] Labs, W. . Y. (2016). R4 - yahoo! movies user ratings and descriptive content information, v.1.0. Accessed 11 May 2022

Ni, J., Li, J., and McAuley, J. (2019). Justifying recommendations using distantly-labeled reviews and fine-grained aspects. In *Proceedings of the 2019 Conference on Empirical Methods in Natural Language Processing and the 9th International Joint Conference on Natural Language Processing (EMNLP-IJCNLP)* (Hong Kong, China: Association for Computational Linguistics), 188–197. doi:10.18653/v1/D19-1018

Rimaz, M. H., Hosseini, R., Elahi, M., and Moghaddam, F. B. (2021). Audiolens: Audio-aware video recommendation for mitigating new item problem. In *Service-Oriented Computing – ICSOC 2020 Workshops*, eds. H. Hacid, F. Outay, H.-y. Paik, A. Alloum, M. Petrocchi, M. R. Bouadjenek, A. Beheshti, X. Liu, and A. Maaradji (Cham: Springer International Publishing), 365–378

[Dataset] Rohrbach, A., Rohrbach, M., Tandon, N., and Schiele, B. (2015). A dataset for movie description

Roy, S. and Guntuku, S. C. (2016). Latent factor representations for cold-start video recommendation. In *Proceedings of the 10th ACM Conference on Recommender Systems* (New York, NY, USA: Association for Computing Machinery), RecSys '16, 99–106. doi:10.1145/2959100.2959172

[Dataset] Sun, T. and Yang, C. (2021). Iptv dataset. doi:10.21227/wqy2-h486

Wu, S., Rizoiu, M.-A., and Xie, L. (2019). Estimating attention flow in online video networks. *Proc. ACM Hum.-Comput. Interact.* 3. doi:10.1145/3359285

Xu, J., Mei, T., Yao, T., and Rui, Y. (2016). Msr-vtt: A large video description dataset for bridging video and language. In *2016 IEEE Conference on Computer Vision and Pattern Recognition (CVPR)*. 5288–5296. doi:10.1109/CVPR.2016.571
